# The Use of High-Flow Nasal Cannula and Non-Invasive Mechanical Ventilation in the Management of COVID-19 Patients: A Prospective Study

**DOI:** 10.3390/v15091879

**Published:** 2023-09-05

**Authors:** Sumalatha Arunachala, Ashwaghosha Parthasarathi, Chetak Kadabasal Basavaraj, Sowmya Malamardi, Shreya Chandran, Hariharan Venkataraman, Mohammed Kaleem Ullah, Koustav Ganguly, Swapna Upadhyay, Padukudru Anand Mahesh

**Affiliations:** 1 Department of Respiratory Medicine, JSS Medical College, JSS Academy of Higher Education and Research, Mysuru 570015, India; a.suma86@gmail.com (S.A.); drchetak@gmail.com (C.K.B.); dr.sony.malamardi@gmail.com (S.M.); shreyachandran@gmail.com (S.C.); harihar5597@gmail.com (H.V.); 2 Public Health Research Institute of India, Mysuru 570020, India; 3 Allergy, Asthma, and Chest Centre, Krishnamurthy Puram, Mysuru 570004, India; ap2320@rwjms.rutgers.edu; 4 RUTGERS Centre for Pharmacoepidemiology and Treatment Science, New Brunswick, NJ 08901, USA; 5 School of Psychology & Public Health, College of Science Health and Engineering, La Trobe University, Melbourne, VIC 3086, Australia; 6 Centre for Excellence in Molecular Biology and Regenerative Medicine (A DST-FIST Supported Center), Department of Biochemistry (A DST-FIST Supported Department), JSS Medical College, JSS Academy of Higher Education and Research, Mysuru 570015, India; ka7eem@jssuni.edu.in; 7 Division of Infectious Disease and Vaccinology, School of Public Health, University of California, Berkeley, CA 94720, USA; 8 Unit of Integrative Toxicology, Institute of Environmental Medicine (IMM), Karolinska Institute, 17177 Stockholm, Sweden; koustav.ganguly@ki.se

**Keywords:** COVID-19, pneumonia, ARDS, high-flow nasal cannula, non-invasive mechanical ventilation

## Abstract

High-flow nasal cannula (HFNC) and ventilator-delivered non-invasive mechanical ventilation (NIV) were used to treat acute respiratory distress syndrome (ARDS) due to COVID-19 pneumonia, especially in low- and middle-income countries (LMICs), due to lack of ventilators and manpower resources despite the paucity of data regarding their efficacy. This prospective study aimed to analyse the efficacy of HFNC versus NIV in the management of COVID-19 ARDS. A total of 88 RT-PCR-confirmed COVID-19 patients with moderate ARDS were recruited. Linear regression and generalized estimating equations (GEEs) were used for trends in vital parameters over time. A total of 37 patients were on HFNC, and 51 were on NIV. Patients in the HFNC group stayed slightly but not significantly longer in the ICU as compared to their NIV counterparts (HFNC vs. NIV: 8.00 (4.0–12.0) days vs. 7.00 (2.0–12.0) days; *p* = 0.055). Intubation rates, complications, and mortality were similar in both groups. The switch to HFNC from NIV was 5.8%, while 37.8% required a switch to NIV from HFNC. The resolution of respiratory alkalosis was better with NIV. We conclude that in patients with COVID-19 pneumonia with moderate ARDS, the duration of treatment in the ICU, intubation rate, and mortality did not differ significantly with the use of HFNC or NIV for respiratory support.

## 1. Introduction

High-flow nasal cannula (HFNC) and non-invasive ventilation (NIV) are used for treating mild to moderate acute respiratory distress syndrome (ARDS). HFNC delivers oxygen at high rates through a nasal interface, creating positive end-expiratory pressure (PEEP) to reduce dead space, eliminate carbon dioxide, and prevent lung collapse [1]. It allows patients to maintain natural breathing even when self-prone, making it effective for ARDS [2]. NIV, administered via a mask or helmet with portable devices or ventilators, enhances gas exchange and reduces breathing effort [3,4].

HFNC and NIV were extensively used during the COVID-19 pandemic for the treatment of mild to moderate ARDS. The majority of the trials during this period have studied mortality rates and the need for invasive mechanical ventilation [5,6,7,8,9,10,11]. Others studied the length of ICU stay, safety issues like aerosol generation, comfort, days free of respiratory support, the incidence of pneumothorax and pneumomediastinum, and 90-day mortality [12,13,14,15,16,17]. Evidence on safety, aerosol generation, pneumothorax, and oxygenation is robust, with both NIV and HFNC showing similar characteristics. However, studies on mortality are conflicting. Many studies showed clinical equipoise in terms of mortality and the need for invasive mechanical ventilation [5,10,11,12]. However, others showed higher mortality with NIV [13,18,19], whereas one study reported higher mortality with HFNC [9]. This difference in treatment effect may be due to the involvement of severe ARDS patients [11], variations in the mode of NIV used [8], small sample size [5,6], differences in the time from symptom onset to hospitalization [6,9], differences in SOFA scores [6,9], and difference in time from hospitalization to start of NIV or HFNC [8,9]. A systematic review and meta-analysis revealed higher mortality associated with NIV administered via a facemask compared to HFNC, but intriguingly, mortality rates were similar to HFNC when NIV was used with the helmet interface [19]. There was no difference between NIV and HFNC regarding intubation rates, oxygenation (PaO_2_/FiO_2_), length of ICU stay, length of hospital stay, and days free from invasive mechanical ventilation. However, the systematic review had several limitations. Although the meta-analysis included 23 studies, many were observational in nature, with a limited number of RCTs (3 RCTs, 8 prospective studies, and 12 retrospective studies) and sample sizes as low as 30 patients. The patient population was heterogeneous and included mild, moderate, and severe ARDS, and no sub-group analysis was conducted for varying severity of ARDS. These patients had different levels of Pressure Support and PEEP, which were not considered in the analysis. The interface used also varied (4 studies reported using a helmet, 11 studies used a face mask and 7 studies did not report the interface used). Also, there were limited studies from LMICs. Hence, more original studies, especially from LMICs using NIV in a specified population of ARDS, are required to generate robust evidence and to compare the efficacy of HFNC versus NIV in moderate COVID-19 ARDS.

The pandemic overburdened healthcare systems worldwide, and many countries lacked the surge capacity required to handle the spike of COVID-19 patients who required advanced life support [20]. Due to inadequate healthcare delivery infrastructures, a lack of critical care units and mechanical ventilators, and a high incidence of comorbid conditions, the pandemic has significantly impacted low and middle-income countries (LMICs) [20,21]. There is, however, still a paucity of data regarding the efficacy of HFNC compared to NIV in COVID-19 patients, especially from LMIC countries. Since using HFNC can help relieve some of the strain on healthcare resources, narrowing this knowledge gap is crucial.

As a result, we carried out prospective observational research to assess the outcomes of COVID-19 patients with acute hypoxemic respiratory failure brought on by ARDS who were administered either ventilator NIV or HFNC after being admitted to the intensive care unit.

## 2. Materials and Methods

This prospective observational study was conducted in a multidisciplinary ICU of a tertiary care university teaching hospital in South India. RT-PCR-confirmed COVID-19 patients with acute hypoxemic respiratory failure due to moderate ARDS between September 2020 and November 2020 were included after obtaining institutional ethical committee approval (Approval number: JSSMC/IEC/141020/09 NCT/2020–2021).

### 2.1. Inclusion Criteria

Patients were screened for COVID-19 using RT-PCR (Rotor-gene, MDX 5plex, Qiagen, Venlo, Netherlands). RT-PCR-confirmed COVID-19 patients who developed acute hypoxemic respiratory failure due to moderate ARDS and received either high-flow nasal cannula or non-invasive ventilation as initial therapies were included.

Criteria for starting NIV/HFNC included the use of accessory muscles of respiration with RR > 25 cycles per minute with a P/F ratio < 200 mm of hg while breathing on oxygen of 10 litres/minute, as per the National Institute of Health COVID-19 guidelines [22]. Irrespective of any switchover to either therapy at a later stage, the patients were grouped according to the initial respiratory support they received and analysed accordingly.

### 2.2. Exclusion Criteria

Exclusion criteria included patients who received only oxygen throughout their ICU stay, patients requiring urgent invasive mechanical ventilation due to altered sensorium or severe ARDS, those missing data necessary for analysis, patients opting for palliative care, PaCO_2_ > 45 mm of hg, hemodynamic instability, Glasgow coma scale <12, facial injuries preventing the application of a face mask or nasal cannula, pregnancy, metabolic acidosis with pH < 7.30, and patients who underwent recent surgery (<7 days).

In a predetermined format, the following data were retrieved from the patients’ files: demographics, baseline haematology, renal function tests, arterial blood gases, vital signs, presence or absence of septic shock and multiorgan dysfunction syndrome (MODS), and Acute Physiology and Chronic Health Evaluation II (APACHE II) score at the time of admission. Included patients used either HFNC or NIV as initial therapy.

### 2.3. Settings for HFNC

HFNC was administered by AIRVO 2(Fisher and Paykel, Auckland, New Zealand) HFNC machine. The flow was kept between 30 and 60 L/min according to the patient’s condition, with a temperature between 34 and 37 °C. The fraction of inspired oxygen (FiO_2_) was adjusted to keep the peripheral blood oxygen saturation (SpO_2_) between 88 and 92%. Close monitoring of the vital signs and arterial blood gases was performed continuously. If HFNC was not successful (worsening dyspnoea, SpO_2_ levels less than 88%, or worsening respiratory acidosis), then NIV was started if endotracheal intubation was not required. A switch to NIV was defined as the failure of HFNC.

### 2.4. Settings for NIV

Non-invasive ventilation was provided with a ventilator (Servo-I, Maquet ventilator, Goteborg, Sweden) using a face mask of appropriate size for the patient. Pressure support with positive end-expiratory pressure was the mode used, with initial inspiratory pressure between 8 and 10 cm H_2_O to generate a tidal volume of a minimum of 6 mL/kg body weight, positive end-expiratory pressure set at a minimum of 5 cm of H_2_O and further titrated to clinical response, and FiO_2_ adjusted to target SpO_2_ of 88–92%. Settings were continuously adjusted according to the clinical response. In case of worsening symptoms and failure in maintaining the oxygenation at the desired levels or intolerance to NIV, we used HFNC if mechanical ventilation was not warranted. A switch to HFNC was also considered failure of NIV.

NIV and HFNC were considered failures when intubation and invasive mechanical ventilation were performed. Vitals were monitored serially at admission, 6 h, and 12 h to detect failure of respiratory therapies, and Indian Society of Critical Care Medicine (ISCCM) guidelines were followed for indications for invasive mechanical ventilation [23]. Treatment of COVID-19 patients was standardized with protocolized care, including steroids and prophylactic anticoagulation as per government of India guidelines, which were updated regularly on the Ministry of Health and Family Welfare (MOHFW) website [24].

### 2.5. Statistical Analysis

Statistical analysis was performed using Jamovi (v1.6, The Jamovi project, SYD, AUS). The extracted data were first tested for normality using the Shapiro–Wilk test. Continuous variables were presented as mean ± standard deviation if the data were normally distributed. If the data were not normally distributed, they were presented as medians with their interquartile ranges. Categorical variables were presented as percentages. Statistical significance was assessed using Student’s “t” or the Wilcoxon test for continuous variables, depending on the normality of the distribution of data. For categorical variables, Pearson’s chi-square test was employed.

The Kaplan–Meier method was used to draw up 28-day survival curves, while the survival rates were compared using the log-rank test. Cox proportional hazards regression analyses were used to identify independent variables associated with mortality outcomes. Variables presumed to be of clinical importance were included in the model. A two-tailed *p*-value of <0.05 was considered statistically significant.

To address the vital factors behaviour overall, HFNC and NIV groups were separately regressed on vital parameters: respiratory rate (RR), heart rate (HR), P/F ratio and PaCO_2_, age, and gender using a Poisson regression model.

To observe the linearity trends in the vital parameters, model-based changes were estimated. We also visually examined the time trends. To understand the nature of the vital parameters, a generalized estimation equation (GEE) approach was used. We assumed a stronger correlation between the current vital measures and the recent vital measure than the previous time points; first order auto-regression was chosen as the correlation matrix. In the model predictions, all data were operationalized as the count data and analysed using the negative binomial regression with log link. As the vital parameters were measured repeatedly across multiple periods, we used GEE; this model is robust and accounts for consistent parameter estimates. Also, the trends of association, confidence intervals, and *p*-values were reported for the estimates.

## 3. Results

A total of 88 COVID-19-positive patients were recruited for the study. Of these, 37 (42.02%) patients were on high-flow nasal cannula (HFNC) and 51 (57.95%) patients were on non-invasive ventilation (NIV) as the first-line intervention. The mean age was 61.5 (54.9–69.1) in the HFNC group and 55.0 (45.0–66.0) in the NIV group. There was no significant difference between the groups regarding age (*p* = 0.172). The HFNC group consisted of 30 (81.1%) males while the NIV group consisted of 40 (80.4%), with no significant difference between the groups (*p* = 0.830). The patients in the HFNC group stayed slightly but not significantly longer in the ICU as compared to their NIV counterparts (HFNC vs. NIV: 8.00 (4.0–12.0) days vs. 7.00 (2.0–12.0) days; *p* = 0.055).

**Figure 1:**
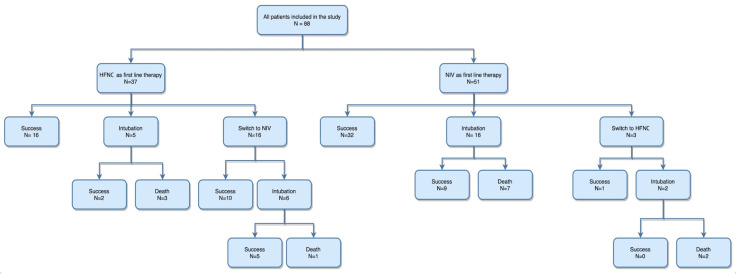
Flowchart showing the outcomes in both HFNC and NIV groups.

Among the 37 patients who received HFNC as their first-line intervention, 16 required no further respiratory support escalation. Five patients were taken up for endotracheal intubation due to progressive respiratory decompensation, three of whom passed away, while the other two required no escalation. The remaining 17.8% of the HFNC group were shifted to NIV for further respiratory support, of which six patients required intubation. One patient who was intubated passed away, while the rest required no escalation of respiratory support. Among the 51 patients who were initially started on NIV treatment, 32 had a favourable outcome, 16 worsened and needed intubation and mechanical ventilation, and 3 were switched to HFNC. Of the intubated patients, nine had a favourable outcome, while two of the patients who were shifted to HFNC were taken up for intubation, after which neither patient had a favourable outcome (Figure 1). The intubation rate was 29.7% and 33.3%, while the mortality rate was 10.8% and 17.6% in the HFNC and NIV groups, respectively. This result, however, was not statistically significant (Table 1). The differences in duration of stay in the ICU between the two groups were also not statistically significant (*p* = 0.63).

APACHE II score and systolic blood pressure were marginally higher in the NIV group, and the diastolic blood pressure was slightly higher in the HFNC group (Table 1). The prevalence of hypertension was higher in the HFNC group, while that of chronic heart disease, chronic respiratory disease, and other comorbidities was higher in the NIV group. However, this difference was not statistically significant (Table 1). Haemoglobin, TLC, platelet count, NLR, blood urea, serum creatinine, potassium, and chloride levels did not show any statistically significant differences between the groups (Table 1).

### 3.1. Results of Statistical Analysis for Success/Failure of HFNC and Ventilator NIV

The respiratory rate, PaO_2_/FiO_2_, PaCO_2_, and heart rate showed significant improvements at 6 and 12 h post-admission when a within-group comparison was performed (Table 2).

The results of the Poisson regression of various vital factors are shown in Table 3. As discussed, the mean values of the vital parameters revealed that the more pronounced changes in respiratory rate, heart rate, and PaO_2_/FiO_2_ took place between baseline and further follow-ups every 6th hour or by the end of the 12th hour among subjects with HFNC therapy. With the increase in time, respiratory rate and heart rate declined in subjects with HFNC therapy (Beta coefficient −β = −0.11; 95% CI, −0.16, −0.07; *p* = 0.0001) (β = −0.029; 95% CI, −0.05, −0.006; *p* = 0.013) and an increase in PaO_2_/FiO_2_ ratio was noted (β = 0.0042; 95% CI, 0.0005, −0.008; *p* = 0.026). In subjects treated with NIV, the respiratory rate declined (Beta coefficient −β = −0.05; 95% CI, −0.08, −0.024; *p* = 0.002), while PaCO_2_ and PaO_2_/FiO_2_ observed a significant increase over time. Meanwhile, PaCO_2_ had no variations over time in HFNC-treated subjects.

**Figure 2:**
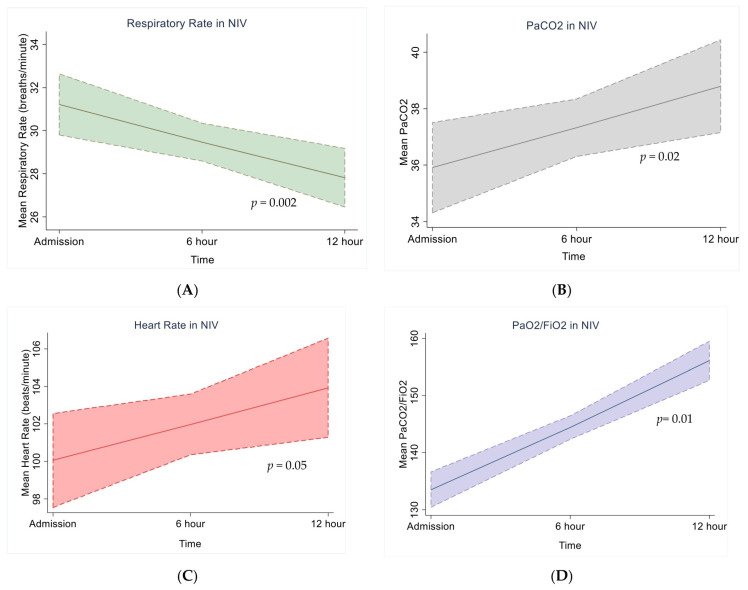
Trends in RR, HR, P/F ratio, and PaCO_2_ over time for NIV. Note: RR = respiratory rate in breaths per minute, HR = heart rate (beats per minute), P/F ratio = partial pressure of oxygen to fraction of inspired oxygen ratio, and PaCO_2_ = partial pressure of carbon dioxide in arterial blood in mmHg. Vital signs (**A**) RR (**B**) HR (**C**) P/F ratio (**D**) PaCO_2_ at the start of the NIV are recorded and reported on admission, 6th hour, and 12th hour on the horizontal *x*-axis, adjusted for age and gender.

**Figure 3:**
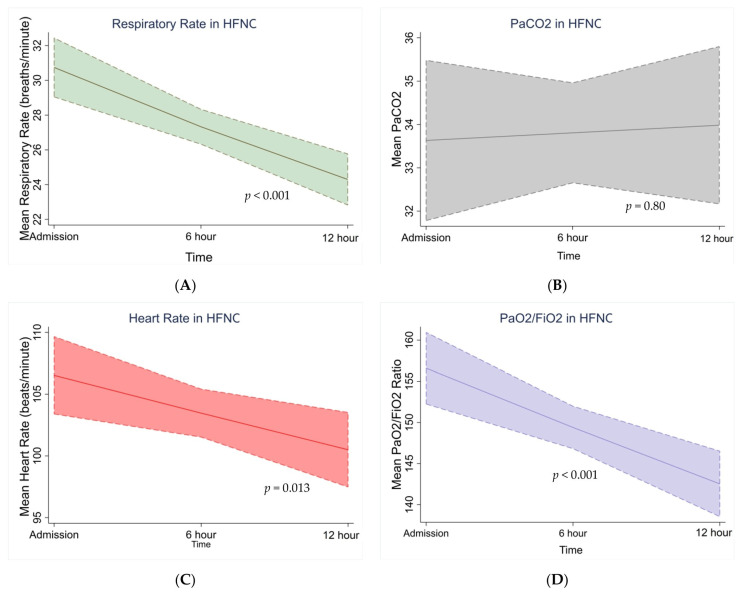
Trends in RR, HR, P/F ratio, and PaCO_2_ over time for HFNC. Note: RR = respiratory rate in breaths per minute, HR = heart rate (beats per minute), P/F ratio = partial pressure of oxygen to fraction of inspired oxygen ratio, and PaCO_2_ = partial pressure of carbon dioxide in arterial blood in mmHg. Vital signs (**A**) RR (**B**) HR (**C**) P/F ratio (**D**) PaCO_2_ at the start of the NIV are recorded and reported on admission, 6th hour, and 12th hour on the horizontal *x*-axis, adjusted for age and gender.

The model-based predictions for the four repeat measures of vital parameters over three time points observed a sustained decline in the respiratory rate in both HFNC and NIV; observations in other vital parameters varied (Figure 2 and Figure 3). Such a decline was also observed with HR in subjects treated with HFNC, but it increased in subjects with NIV therapy. PaCO_2_ also increased among NIV subjects. However, such significant differences were not observed in HFNC subjects (Figure 2 and Figure 3). PaO_2_/FiO_2_ increased in both HFNC and NIV subjects.

### 3.2. The Role of Vital Parameters in the Successful Outcome of Either Ventilation Therapy

Successful outcome here is defined as the absence of need for invasive mechanical ventilation and patient survival at the time of the discharge.

**Figure 4:**
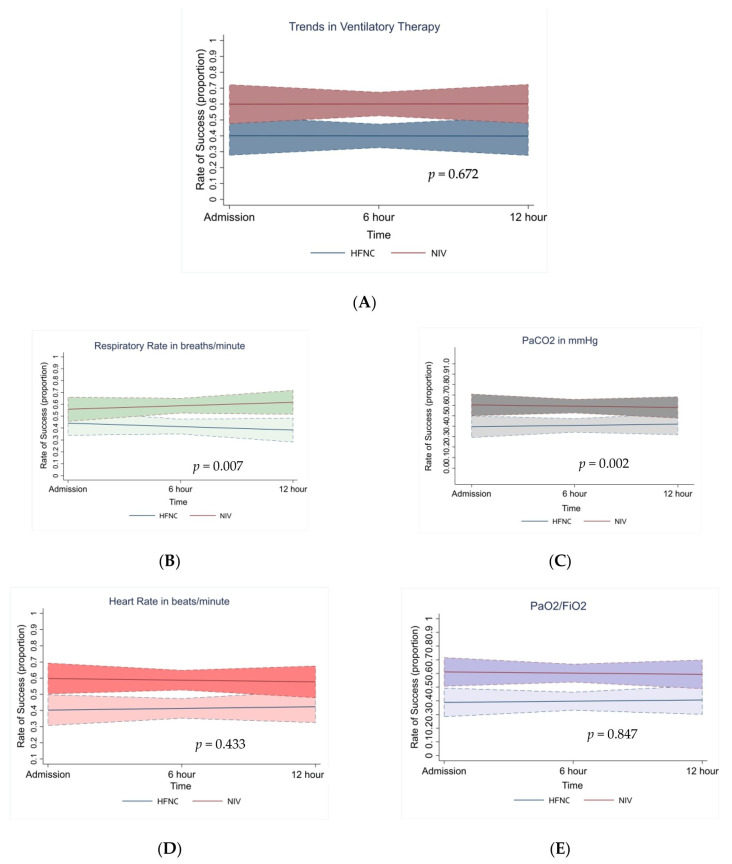
(**A**) Overall rate of success in HFNC and NIV ventilator therapy over the initial hours on admission. The rate of success with respect to each of the vital parameters: (**B**) RR, (**C**) PaCO_2_, (**D**) HR, and (**E**) PaO_2_/FiO_2_ ratio. Note: Proportion 0.1 = 10%, 1 = 100%. In each of the plot areas (**B**–**E**), dark colour represents the group of subjects with NIV therapy (n = 32) and light colour represents HFNC therapy (n = 27); the slope is represented by the red and blue line for NIV and HFNC group, respectively. Note: All the above figures are adjusted for age and gender characteristics.

The overall rate of success in NIV was slightly higher (38.10%) compared to HFNC (32.14%), but this was not statistically significant (Figure 4A). The respiratory rate and PaCO_2_ in both therapies were associated with the improved rate of success [β = 0.06, 95% CI (0.017, 0.115); *p* = 0.007] and [β = 0.05, 95% CI (0.018, 0.084); *p* = 0.002]; no significant associations were found for heart rate and P/F ratio (Figure 4B–E). With GEE analysis, the associations with the rate of success were similar for both HFNC and NIV when modelling the repeat estimates and were consistent over time. In our patients, change in vital parameters like heart rate and P/F ratio in either therapy had no influence on the rate of success (patient survival).

### 3.3. Results of Statistical Analysis for Mortality Outcomes in HFNC and Ventilator NIV

**Figure 5:**
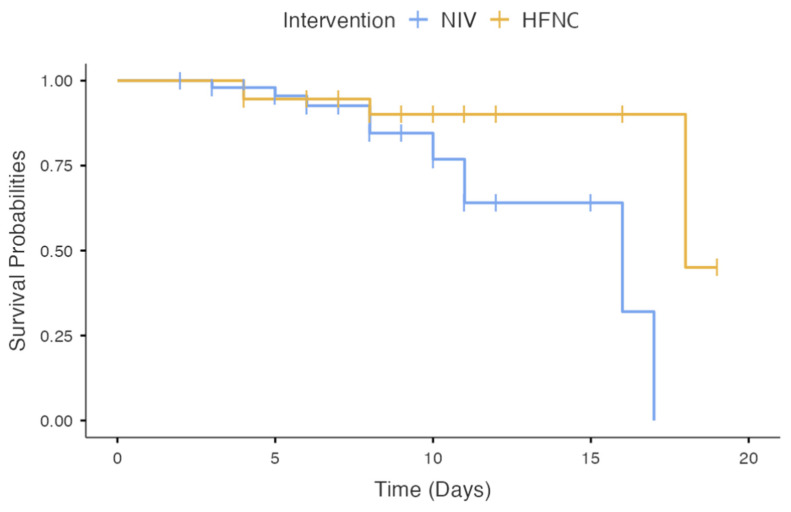
Shows 28-day survival probabilities in patients with HFNC and NIV as first-line interventions.

The Kaplan–Meier analysis performed to assess 28-day survival probabilities in patients with HFNC and NIV as first-line interventions showed no significant difference between the two groups (Log-rank: *p* = 0.22) (Figure 5).

Univariate Cox regression analysis found APACHE II scores, the presence of complications, the presence of chronic heart disease, and the presence of chronic respiratory disease to have significantly high hazard ratios. However, upon multivariate analysis, only APACHE II scores (HR (95% CI): 1.32 (1.06–1.64); *p* = 0.013), presence of chronic heart disease (HR (95% CI): 1.42 (1.08–1.88); *p* = 0.013), and chronic respiratory disease (HR (95% CI): 5.18 (1.45–18.46); *p* = 0.011) were found to be strong independent predictors of mortality (Table 4).

## 4. Discussion

The present study evaluated the hypothesis that NIV delivered via ventilators (V-NIV) and HFNC have comparable success and mortality outcomes in patients with hypoxemic respiratory failure secondary to COVID-19 pneumonia and moderate ARDS. Patients on HFNC and V-NIV had similar changes in parameters like RR and PaO_2_/FiO_2_. Although improvement in respiratory rates over time was seen in both groups, it was better in the HFNC group compared to the V-NIV groups. PaCO_2_ was normalised in patients with V-NIV compared to the HFNC group. Variations in HFNC and V-NIV success rates (survival and successful shift out of ICU without requiring invasive mechanical ventilation) were similar in both subgroups. Patients treated with V-NIV had similar outcomes concerning mortality and the need for endotracheal intubation compared to the HFNC group. These results support our hypothesis that NIV delivered via a ventilator and HFNC have comparable outcomes in COVID-19 patients with moderate ARDS. Our study has also confirmed that patients having higher APACHE, comorbidities with chronic heart failure, and chronic respiratory failure were at risk of higher mortality in COVID-19 disease.

Patients’ respiratory rates on admission and decrease in respiratory rates over the first 12 h were a better predictor of the success of therapy when compared with other parameters, regardless of whether V-NIV or HFNC was used. Additionally, in patients with V-NIV therapy, we observed that normalisation of PaCO_2_ was a key predictor of the success of the therapy. Under physiological conditions, the rise in PaCO_2_ represents hypoventilation. However, in patients with hypoxemia, there is a fall in PaCO_2_ due to hypoxemia-induced alveolar hyperventilation. PaCO_2_ returns to the normal range once the hypoxemia settles. Thus, in our patients, the normalisation of PaCO_2_ may be due to the resolution of hypoxemia and suggests early responsiveness of hypoxia in these COVID-19 patients. This normalisation of PaCO_2_ was not seen in the first 12 h with HFNC. The other vital parameters had similar responses in either of the ventilation therapies.

Although HFNC and NIV are comparable in stable hypercapnic COPD and pulmonary fibrosis patients when delivered over short periods [25,26,27], the use of NIV in acute hypoxemic respiratory failure has not had convincing evidence so far, and there was an increased risk of mortality when using NIV compared to HFNC in these patients [3,28,29]. On the contrary, a recent large cohort trial evaluating trends of NIV use has not shown increased mortality with the use of NIV in acute hypoxemic respiratory failure [3]. One of the important reasons for these discrepancies relates to whether NIV was delivered via a ventilator or through portable machines. Some observational studies compared HFNC and NIV in COVID-19 pneumonia with ARDS; each measured different outcomes [5,7]. Both studies have found similar mortality rates between the HFNC group and NIV group in patients with COVID-19 pneumonia with ARDS. Our study adds to the limited body of evidence that using NIV via ventilator or using HFNC may lead to similar mortality risk in acute hypoxemic respiratory failure.

So far, there are only two randomized controlled trials, conducted by Nair PK et al. [10] and Greico L et al. [12], on NIV compared to HFNC in patients with moderate to severe ARDS due to COVID-19 pneumonia. Both studied different primary outcomes. Nair et al. [10] studied HFNC and NIV failure rates in COVID-19 pneumonia with severe ARDS leading to the requirement of invasive mechanical ventilation and mortality. There was no statistically significant difference in intubation rates in HFNC and NIV groups, as well as no significant differences in mortality between HFNC and NIV, which is similar to our study observations. Greico et al. [12] studied respiratory-support-free days as the primary outcome in moderate and severe ARDS due to COVID-19 pneumonia. However, the need for intubation was significantly lower with helmet NIV compared to HFNC in the RCT, which is different from our study observations. This could be due to differences in the interface used by Grieco et al. Nevertheless, 28-day and 60-day mortality, ICU mortality, and in-hospital mortality were similar between both groups, which is similar to the results of the present study (Table 5).

Conversely, a large multicentric prospective cohort study by the COVID-ICU group [13] involving 137 centres in France which evaluated different non-invasive strategies in COVID-19 pneumonia with ARDS showed increased mortality with the use of NIV. Nonetheless, out of 556 patients who received non-invasive oxygenation strategies, only 34 received NIV alone; 481 patients received HFNC alone, whereas another 41 patients received combined NIV and HFNC for respiratory support. Due to the small number of subjects receiving NIV alone and the non-randomized nature of the study design, which has the potential for significant bias, the findings of this study cannot be taken as evidence against the use of NIV in acute hypoxemic respiratory failure. Costa WNS et al. [6] evaluated the safety and outcomes of 39 COVID-19 patients treated with NIV and HFNC in a retrospective study. Similar to our study, they did not find any difference in mortality, need for invasive mechanical ventilation, or increased duration of ICU stay between the two groups. However, the study findings may not be generalizable to moderate to severe COVID-19 patients, as the mean FiO_2_ used was 38%, suggestive of milder COVID illness. Recently, a systematic review and meta-analysis by Yun Peng et al. [19], including 3 RCTs and 20 observational studies comparing the use of NIV versus HFNC in both ICU and ward patients, demonstrated no difference in mortality rate between helmet NIV and HFNC, confirming the findings of our study.

The findings of our study add support to the hypothesis that the use of HFNC and NIV in patients with acute hypoxemic respiratory failure due to COVID-19 pneumonia with moderate ARDS may have a similar outcome in terms of the need for mechanical ventilation and hospital mortality, but further larger studies are needed to confirm these findings. However, from an economic perspective, HFNC is less expensive (HFNC costs USD 2675–3125 compared to the average cost range of USD 12,500 to USD 62,500 for a ventilator NIV). HFNC is easy to maintain and has a low daily running cost. Although the disposable circuit of HFNC adds to the one-time circuit cost (USD 125), it can be used for the entire duration of a hospital stay. HFNC is less labour-intensive and does not require expert nursing care or respiratory therapists to troubleshoot, unlike V-NIV. Patient compliance is also better due to the comfortable nasal cannula compared to the face mask which is used in V-NIV. HFNC can also be used in non-ICU settings, where there is less intensive monitoring. These features make HFNC an attractive option in low- and middle-income countries, especially during times of pandemic. Thus, based on our study findings and the above factors, HFNC may be preferentially used over ventilator NIV in LMIC countries. None of the studies mention whether the NIV used in the studies was ventilator NIV or portable NIV. There is a felt need to compare portable NIV, which is even more economical, with ventilator NIV and HFNC.

Most of the trials conducted so far (including our study) have shown a considerable switch over from HFNC to NIV or vice-versa. Previous studies have also reported a switch of 17–20% from HFNO to NIV [8,30]. A higher rate of switch was noted in our study. The decision to switch from HFNO to NIV was per clinician discretion. The possible reasons as explained by clinicians were higher PEEP offered by NIV compared to HFNO, which was thought to be beneficial in ARDS as it prevents repeated atelectrauma during tidal breathing, ability to monitor expired tidal volume and thereby minute ventilation, and availability of additional information on ventilator scalars and values like P_0.1_ and RSBI. Serial monitoring of these helped the clinicians understand the trends of patients’ respiratory mechanics better with NIV. A recent RCT by Coudroy et al. [31] compared HFNC and HFNC alternated with NIV in hypoxic immunocompromised patients. The study was underpowered and did not find any difference in mortality between both groups. Thus, there is a lack of evidence on the outcomes with the stacked use of NIV and HFNC. Hence, we suggest that future trials evaluating outcomes with either mode of respiratory support should also consider evaluating the outcomes with a combination therapy of NIV and HFNC.

Our study strengths are that it is one of the few studies comparing NIV and HFNC for COVID-19 pneumonia with ARDS in LMICs, and it is the first to evaluate ventilator NIV or HFNC in moderate ARDS patients. It also evaluated the impact on vital parameters serially over 12 h using advanced statistical methods (GEE). However, limitations include physician bias in treatment decisions, lack of data on awake-prone positioning, bias due to switching between treatments, and limited testing due to resource constraints. 

Due to the small sample size of our study, the generalizability of our results is limited. Though we observed no statistically significant difference in mortality and ICU length of stay, this should be interpreted with caution, and a definitive answer needs a larger sample size, which would provide greater clarity on whether NIV could be superior to HFNC on both mortality and length of ICU stay. This has significant implications during the pandemic, especially in LMICs, as it increases the availability of ICU beds by shifting patients out early and reduces cost.

## 5. Conclusions

In patients with acute hypoxemic respiratory failure due to COVID-19 pneumonia with moderate ARDS, the duration of treatment, intubation rate, and mortality did not differ with the use of HFNC or ventilator NIV for respiratory support. We need further studies to identify whether there are specific characteristics of patients who may have better outcomes with either NIV (ventilator and portable) or HFNC, and there is a need for validation of these findings in large cohorts across different centres.

## Figures and Tables

**Table 1:** Baseline demographic characteristics of the study.

	All Patients (N = 88)	HFNC (N = 37)	NIV (N = 51)	*p*-Value
Age (years)	59.0 (39.7–78.3)	61.5 (54.9–69.1)	55.0 (45.0–66.0)	0.172
Gender				
Male (n, %)	71 (80.7)	30 (81.1)	41 (80.4)	0.830
Female (n, %)	17 (19.3)	7 (18.9)	10 (19.6)	
Duration of ICU stay (days)	8.0 (5.0–12.0)	8.00 (4.0–12.0)	7.00 (2.0–12.0)	0.055
APACHE II score	10.0 (7.8–13.0)	9.0 (7.0–12.0)	11.0 (8.0–13.8)	0.100
Systolic blood pressure	130.0 (120.0–140.0)	132.0 (129.8–150.0)	136.0 (130.0–148.3)	0.840
Diastolic blood pressure	76.0 (70.0–80.0)	80.0 (71.2–88.8)	70.0 (59.9–60.1)	0.338
Complications	18.0 (20.5)	6.0 (16.2)	12.0 (23.5)	0.402
Outcomes				
Success rate (n, %)	48 (54.5)	16 (43.2)	32 (62.7)	0.690
Intubation rate (n, %)	28 (31.8)	11 (29.7)	17 (33.3)	0.720
Mortality rate (n, %)	13 (14.8)	4 (10.8)	9 (17.6)	0.370
Switch to HFNC (n, %)	3 (3.4)	-	3 (5.8)	--
Switch to NIV (n, %)	17 (20.4)	17 (37.8)	-	--
Comorbidities				
Hypertension (n, %)	45 (51.1)	21 (56.7)	24 (47.1)	0.390
Chronic Cardiac disease (n, %)	11 (12.5)	3 (8.1)	8 (15.6)	0.220
Chronic respiratory disease (n, %)	3 (3.4)	1 (2.7)	2 (3.9)	0.230
Others (n, %)	11 (12.5)	3 (8.1)	8 (15.6)	0.610
Haematological investigations				
Haemoglobin	12.82 ± 1.96	13.13 ± 1.81	12.60 ± 2.05	0.211
Total Leukocyte Count	10.10 (4.67–15.53)	9.700 (7.60–13.07)	11.54 (8.08–13.19)	0.067
Platelet	2.875 (1.90–3.85)	2.90 (2.50–3.20)	3.0 (2.40–3.60)	0.100
NLR	11.28 (0.61–21.89)	11.40 (8.80–15.0)	12.00 (6.00–17.70)	0.478
Urea	43.00 (23.25–62.75)	43.0 (36.0–48.7)	45.0 (32.30–59.30)	0.087
Creatinine	0.810 (0.53–1.09)	0.80 (0.70–0.90)	0.80 (0.70–1.00)	0.279
Sodium	137.00 (131.25–142.75)	136.0 (132.0–140.0)	138 (132.12–143.88)	0.018
Potassium	4.35 (3.65–5.05)	4.40 (3.90–4.70)	4.40 (4.10–4.70)	0.576
Chloride	97.00 (92.25–101.75)	96.47 ± 3.10	97.97 ± 5.18	0.122

ICU: intensive care unit; APACHE II: Acute Physiology and Chronic Health Evaluation; HFNC: high-flow nasal cannula; NIV: non-invasive ventilation; NLR: Neutrophil-to-Lymphocyte Ratio.

**Table 2:** Vital signs and blood gas analysis of the study population on admission and 6 h and 12 h after treatment.

	At Admission	After 6 h	After 12 h	*p*-Value
HFNC				
RR	31.14 (22–42)	26.65 (21–38)	24.68 (19.0–38)	<0.01
PaCO_2_	33.47 (22–61)	34.26 (24.9–54)	33.97 (23–52)	0.04
HR	106.97 (74–150)	102.65 (64–162)	100.94 (52–150)	0.02
PaO_2_/FiO_2_	134.38(58–241.5)	144.38 (61–262.7)	157.90 (61–300)	0.03
NIV				
RR	31.46 (21–50)	29.04 (22.0–46.0)	28.10 (22.0–48.0)	0.03
PaCO_2_	35.47 (18–81)	38.30 (25–74)	38.34 (22–69)	0.04
HR	100.80 (46–200)	100.49 (46–148)	104.95 (46–158)	<0.01
PaO_2_/FiO_2_	134.38 (58–241.5)	145.86 (61–262.5)	156.53 (61–300)	<0.01

RR: respiratory rate (breaths/minute); PaCO_2_: partial pressure of alveolar carbon dioxide (mmHg); HR: heart rate (beats/minute); PaO_2_/FiO_2_: partial pressure of oxygen to fraction of inspired oxygen ratio.

**Table 3:** The estimates for the vital parameters recorded over time in patients treated with HFNC and NIV.

	HFNC (N = 37)		NIV (N = 51)	
	Unadjusted Beta Co-Efficient (β)	Adjusted Beta Co-Efficient (aβ)	Unadjusted Beta Co-Efficient (β)	Adjusted Beta Co-Efficient (aβ)
RR	−0.11 (−0.16, −0.07) ***	−0.11 (−0.16, −0.07) ***	−0.05 (−0.09, −0.02) **	−0.05 (−0.09, −0.02) **
PaCO_2_	0.007 (−0.03, 0.04)	0.005 (−0.03, 0.04)	0.03 (0.004, 0.071) *	0.03 (0.004, 0.072) *
HR	−0.029 (−0.05, −0.006) *	−0.029 (−0.05, −0.006) *	0.02 (0.0004, 0.039) *	0.01 (−0.0006, 0.038)
PaO_2_/FiO_2_	0.0042 (0.0005, 0.0080) *	0.0044 (0.0005, 0.0083) *	0.053 (0.035, 0.071) ***	0.054 (0.036, 0.072) ***

Note: Adjusted for age and gender over time periods on admission, 6 hourly, 12 hourly; * = *p* < 0.05, ** = *p* < 0.01, *** = *p* < 0.001; RR: respiratory rate (breaths/minute); PaCO_2_: partial pressure of alveolar carbon dioxide (mmHg); HR: heart rate (beats/minute); PaO_2_/FiO_2_: partial pressure of oxygen to fraction of inspired oxygen ratio.

**Table 4:** Cox regression analysis of risk factors associated with mortality in the study population.

	Characteristic	HR (Univariable)	HR (Multivariable)
Age		1.0 (0.97–1.03)	0.98 (0.94–1.01)
Sex	Male	-	-
	Female	0.58 (0.17–1.92)	0.81 (0.23–2.85)
Intervention	NIV	-	-
	HFNC	0.57 (0.26–1.26)	0.80 (0.32–1.97)
APACHE II		1.31 (1.06–1.61) *	1.32 (1.06–1.64) *
Complications ^†^	Absent	-	-
	Present	5.35 (2.26–12.68) ***	3.02 (0.98–9.25)
Comorbidities			
Hypertension	Absent	-	-
	Present	0.95 (0.44–2.07)	0.98 (0.42–2.32)
Chronic Heart Disease	Absent	-	-
	Present	1.12 (1.05–1.20) ***	1.42 (1.08–1.88) *
Chronic Kidney Disease	Absent	-	-
	Present	1.39 (0.18–10.43)	0.64 (0.02–17.04)
Chronic Respiratory disease	Absent	-	-
	Present	4.24 (1.68–10.69) **	5.18 (1.45–18.46) *

* = *p* < 0.05, ** = *p* < 0.01, *** = *p* < 0.001; HR: hazard ratio; APACHE II: Acute Physiology and Chronic Health Evaluation; HFNC: high-flow nasal cannula; NIV: non-invasive ventilation. ^†^ Complications include but are not limited to multiple organ dysfunction syndrome, failure of intubation, and death.

**Table 5:** Studies comparing outcomes of HFNC and NIV in patients with COVID-19 ARDS in an ICU setting.

Author	Number of Patients	Type of NIV	Outcome			Comments
			Failure and Need for IMV	Ventilator-Free Days	Mortality	
Grieco et al. [12]	109	Ventilator NIV	Lower in the NIV group	Similar in both groups	No significant difference	Patients requiring helmet NIV for >48 h were eventually treated with HFNC, diluting the treatment effect from NIV alone
Nair PR et al. [10]	199	Data not available	Lower in the HFNC group at 7 days	Not available	No significant difference	Single-centre randomized controlled trial did not find the difference between NIV and HFNC
COVID-ICU cohort [13]	556	11 patients out of 34 received portable CPAP. Rest received ventilator NIV	No significant difference	Not assessed between HFNC and NIV groups	Higher mortality in the NIV group	Only 34 patients received NIV. The remaining 484 patients received HFNC
Recovery-RS trial [8]	798	Portable CPAP delivered via NIV device	Lower in the CPAP group	Not assessed	Lower in the CPAP group	The trial was CPAP over HFNC
Costa WNS et al. [6]	37	Portable NIV device	No significant difference	Not assessed	No significant difference	Only 14 patients received NIV and the majority received HFNC
Our study	88	Ventilator NIV	No significant difference	Not assessed	No significant difference	Performed on moderate ARDS only
Pearson et al. [11]	109	NIV delivered via Helmet	No significant difference	Not assessed	Similar in both groups	Performed on both moderate and severe ARDS
Ranieri et al. [9]	315	Ventilator NIV	Lower in the HFNC group	Not assessed	Similar in both groups	No significant difference in outcome between groups
Shoukri et al. [5]	63	Ventilator NIV	No significant difference	Not assessed	Similar in both groups	No significant difference in outcome between groups

## Data Availability

All data generated or analysed during this study are included in this published article and are available from the corresponding author upon reasonable request.

## Abbreviations

**Abbreviation/Acronym**	**Definition/Full-form**
APACHE II	Acute Physiology and Chronic Health Evaluation
ARDS	Acute Respiratory Distress
CI	Confidence Interval
COVID-19	Coronavirus Disease 2019
CPAP	Continuous Positive Airway Pressure
GEE	Generalized Estimating Equations
HFNC	High-Flow Nasal Cannula
HR	Hazard Ratio
ICU	Intensive Care Unit
ISCCM	Indian Society of Critical Care Medicine
LMICs	Low- and Middle-Income Countries
MODS	Multi-Organ Dysfunction Syndrome
MOHFW	Ministry Of Health and Family Welfare
NIV	Non-Invasive Ventilation
NLR	Neutrophil-to-Lymphocyte Ratio
PaO_2_	Partial Pressure of Oxygen
PaO_2_/FiO_2_	Ratio of Partial Pressure of Arterial Oxygen and Fraction of Inspired Oxygen
PH	Acidity/Alkalinity
RT-PCR	Reverse Transcription Polymerase Chain Reaction
RR	Respiratory Rate
SPO_2_/FIO_2_	Ratio of Oxygen Saturation to The Fraction of Inspired Oxygen
